# Digital Transformation of Korean Articulation and Phonology Assessment: Reliability and Validity of Hi-DongDong

**DOI:** 10.3390/children13101394

**Published:** 2026-10-13

**Authors:** Jaewon Lee, Sejin Kwon, Jin Young Ko, Yulhyun Park, Jinju Lee, Ju Seok Ryu, Jee Hyun Suh

**Affiliations:** 1 Department of Rehabilitation Medicine, Seoul National University Bundang Hospital, Seongnam 13620, Republic of Korearq300@snubh.org (S.K.); jjlee4@snu.ac.kr (J.L.); jseok337@snubh.org (J.S.R.); 2 Department of Rehabilitation, Gyeonggi Provincial Medical Center Anseong, Anseong 17558, Republic of Korea; gpmc7296@medical.or.kr; 3 Department of Rehabilitation, Gyeonggi Provincial Medical Center Icheon, Icheon 17359, Republic of Korea; gpmc7536@medical.or.kr

**Keywords:** speech sound disorders, digital therapeutics, telerehabilitation, assessment instrument, articulation, phonology, validation study

## Abstract

**Highlights:**

**What are the main findings?**

- Hi-DongDong showed excellent inter-rater reliability (ICC ≥ 0.96) and good-to-excellent inter-instrument agreement with UTAP-1/UTAP-2 for utterance-level consonant accuracy indices among 931 enrolled children.
- Validity correlations with UTAP were statistically significant (r = 0.347–0.939), ranging from weak for PVC to strong for consonant accuracy indices, with the closest agreement observed for total PCC and total PCC-R.

**What are the implications of the main findings?**

- Hi-DongDong showed concurrent validity and agreement with UTAP, supporting its potential use in digital and telerehabilitation settings.
- Hi-DongDong may have potential as a screening and monitoring tool but cannot yet replace standardized diagnostic assessment; diagnostic accuracy studies, age-specific normative data, and intra-rater reliability studies are needed before its clinical use.

**Abstract:**

**Background/Objectives**: Speech sound disorders (SSDs) are common in childhood and affect literacy and academic achievement. Standardized assessments such as the Urimal Test of Articulation and Phonology (UTAP) rely on time-intensive manual scoring. We evaluated the reliability and validity of Hi-DongDong, a Delphi-developed digital articulation assessment. **Methods**: This prospective multicenter study across five institutions recruited in total 931 children aged 2–13 years from outpatient clinics and community childcare facilities. Speech samples obtained with Hi-DongDong and UTAP-1 or UTAP-2 were independently evaluated by three experienced speech–language pathologists. Analyses were conducted on participants with valid paired datasets, using intraclass correlation coefficients (ICCs), Pearson correlations, Wilcoxon signed-rank tests, and Bland–Altman analyses. **Results**: Inter-rater reliability was excellent (ICC ≥ 0.96). Inter-instrument reliability was good to excellent for total percentage of correct consonants (PCC) and total PCC–revised. All outcomes correlated significantly with UTAP (r = 0.347–0.939, *p* < 0.001). Utterance-level consonant accuracy indices showed minimal mean bias, whereas phonological mean length of utterance, vowel accuracy, and word accuracy showed systematic bias or wide limits of agreement, precluding interchangeable use. **Conclusions**: Hi-DongDong showed excellent inter-rater reliability and concurrent validity, particularly for consonant accuracy; however, its diagnostic accuracy remains unknown, and it cannot yet replace standardized diagnostic thresholds.

## 1. Introduction

Speech sound disorders (SSDs) refers to difficulties in the perception, motor production, or phonological representation of speech sounds, resulting in reduced speech intelligibility [1]. SSD encompasses both articulation disorders, which involve errors in the physical production of speech sounds, and phonological disorders, which reflect difficulties in the rule-based organization of speech sound systems [2]. In South Korea, SSD accounts for approximately 44% of pediatric communication difficulties, with articulation disorders being the most prevalent subtype [3], thereby hindering early literacy and academic achievement. Therefore, early identification and intervention are essential for mitigating long-term developmental risks [4]. Although rehabilitation paradigms have advanced, healthcare disparities persist in marginalized regions. Telerehabilitation provides a transformative strategy to overcome these barriers and optimize efficiency [5]. Accordingly, early diagnosis and timely intervention for SSD through telerehabilitation-based, application-driven articulation assessments are required.

Korean has 19 consonants, with a three-way laryngeal contrast (lenis, aspirated, and fortis) among stops and affricates and a two-way contrast (lenis and fortis) among fricatives; only seven consonants (/p, t, k, m, n, ŋ, l/) occur in syllable-final position, and consonant clusters are not permitted within a syllable [6,7,8]. Contemporary Seoul Korean has seven monophthongs, and diphthongs are formed with the glides /j/ and /w/ [8]. Korean-speaking children generally master vowels by about 3 years of age, whereas consonant acquisition continues into the early school years: most consonants are acquired by 3–4 years of age, while the fricatives /s, s͈/ and the liquid /l/ reach adult-like accuracy only at around 6–7 years [9,10].

The Urimal Test of Articulation and Phonology (UTAP) is the primary standardized instrument for articulation and phonological assessment in South Korea. It is designed to evaluate consonant and vowel production across word positions using a picture-naming task, in which children are prompted to produce target words representing all Korean phonemes in initial, medial, and final positions [11]. Responses were transcribed by trained examiners and scored based on accuracy, yielding measures such as the percentage of correct consonants (PCC) and error pattern profiles (e.g., substitution, omission, distortion) that support phonological analysis and differential diagnosis of SSD [12]. However, manual evaluation remains time-intensive and is subject to variability depending on the examiner’s clinical proficiency. Therefore, recent efforts have focused on the digital transformation of speech sound assessment by integrating artificial intelligence (AI) [13]. However, the digital expansion of UTAP is constrained by licensing restrictions and the inherent difficulty of visualizing onomatopoeic items.

The development of a new assessment instrument generally follows established psychometric procedures, including item development with expert-based content validation, followed by evaluation of measurement properties such as reliability and validity [14]. Accordingly, a novel articulation assessment instrument, “Hi-DongDong,” was developed by 23 experts (12 physiatrists and 11 speech–language pathologists; mean professional experience, 10.69 years) using a three-round modified Delphi process [15]. An initial list of 26 words, drafted on the basis of existing instruments and the literature on word familiarity, was expanded to 35 words to include diphthongs, broaden phonological coverage, and exclude dialectal or unfamiliar words; the expert panel also confirmed that the words were not difficult to visualize or confusing [15]. Regarding the picture stimuli, the illustrations for each target word were created by a professional illustrator to depict the target words clearly and unambiguously and were checked by the research team for correspondence with the target words. However, the illustrations were not formally reviewed by the expert panel during the Delphi process. A quantitative scoring system and standardized assessment procedures were also established [15]. Consequently, this study aimed to evaluate the reliability and validity of Hi-DongDong in children aged 2–13 years.

## 2. Materials and Methods

### 2.1. Study Design

This study adhered to the Guidelines for Reporting Reliability and Agreement Studies (GRRAS). The study protocol was approved by the Institutional Review Board (IRB) of Seoul National University Bundang Hospital (protocol code B-2510-1000-306) on 25 September 2025, before participant enrollment began, and all data were collected prospectively between October and December 2025. The study was registered at ClinicalTrials.gov (NCT07273370) on 19 November 2025, after enrollment had commenced (retrospective registration). All procedures were conducted in accordance with the Declaration of Helsinki. Considering the vulnerability of the pediatric population, written informed consent was obtained from the legal guardians of children aged < 7 years. For children aged ≥ 7 years, written guardian consent and the child’s assent were secured to ensure voluntary participation.

### 2.2. Participant Recruitment and Sample Size Determination

The sample size was determined through an a priori power analysis using G*Power software (version 3.1.9.7) to ensure sufficient statistical power to validate Hi-DongDong. Based on a bivariate normal model for Pearson’s correlation, at least 138 participants were required to detect a medium effect size (r = 0.3; significance level, 0.05; power, 0.95). To maintain methodological rigor consistent with the validation study of UTAP-2 (*n* = 616) [16] and to compensate for a projected 20% attrition rate, the final enrollment target was set at >740 participants.

To ensure generalizability, participants were recruited across five institutions using a competitive enrollment strategy, in which no site-specific quotas were set and all sites recruited concurrently until the overall target sample size was reached. The sampling methodology combined convenience sampling of pediatric outpatients at each site with community-based sampling through direct visits to local childcare facilities, such as daycare centers, within the vicinity of participating institutions.

The inclusion criteria were as follows: (1) children aged 2–13 years; (2) cognitive proficiency sufficient to follow basic instructions; and (3) voluntary participation with informed consent obtained from the child and their legal guardian. The exclusion criteria were as follows: (1) failure to obtain informed consent; (2) moderate-to-severe intellectual disability precluding the ability to complete the assessment procedures; (3) presence of an acute condition, such as respiratory disease, that could temporarily distort acoustic characteristics; and (4) an environment unsuitable for maintaining participant attention or eliciting speech during recording.

Data from the 505 typically developing children assessed with UTAP-2 in this cohort were also used in a separate study examining age-specific agreement between Hi-DongDong and UTAP-2 [17].

### 2.3. Assessment

The assessment protocol and stimuli for Hi-DongDong are detailed in Supplementary Material S1; those for UTAP are described elsewhere [11,16], and are not reproduced owing to copyright restrictions. UTAP-1 refers to the original edition of UTAP [11], and UTAP-2 refers to its revised edition with updated norms [16]; both are full standardized tests rather than shortened versions, and their word-level tests consist of 30 picture-naming items. The Hi-DongDong word set was developed through a three-round modified Delphi process to ensure clinical relevance and phonological representativeness [15]. Candidate items were selected to cover a broad range of Korean phonemes across word positions (initial, medial, and final), with consideration of articulatory features such as manner and place of articulation. The final 35-word list reflects expert consensus on age-appropriate vocabulary, phonological diversity, and suitability for digital administration [15]. Particular emphasis was placed on expanding phonological coverage and reducing item ambiguity, as identified during the Delphi rounds. Consensus in the Delphi process was determined using predefined criteria, including a content validity ratio (CVR) of ≥0.39, a content validity index (CVI) of ≥0.78, a median of ≥3.5, and an interquartile range (IQR) of ≤1.0. Items were retained only when all criteria were satisfied. All seven Korean monophthongs are included in Hi-DongDong, UTAP-1, and UTAP-2; Hi-DongDong additionally includes nine diphthongs, compared with six in UTAP-1 and one in UTAP-2 [11,16]. Compared with UTAP, which uses a fixed standardized item set, the Hi-DongDong word list was specifically designed to enhance phonological coverage and reduce item ambiguity, which may contribute to differences observed in the derived indices.

To minimize pediatric fatigue and isolate discrepancy factors from subjective clinician judgment, consecutive speech recordings were captured in a single session under a standardized protocol across all sites. Within each session, UTAP was administered first, followed by Hi-DongDong; the order of administration was not counterbalanced or randomized. UTAP-2 and UTAP-1 were allocated in a 2:1 ratio for data collection. Each participant’s dataset comprised two discrete sets: 30 stimuli from the assigned UTAP version and 35 stimuli from Hi-DongDong, which were distributed asynchronously to three independent reviewers at an external institution for evaluation.

Rigorous data anonymization and allocation concealment were enforced to prevent potential evaluator bias arising from the distinct word-list compositions of the two instruments. Audio files were coded using non-identifiable alphanumeric labels. All linkages between the two datasets collected from the same participant were severed so that evaluators remained unaware that the sets originated from the same individual. Data assignment order was randomized to neutralize potential expectation bias, facilitating independent acquisition of objective measurements for each instrument.

Assessments were performed by three speech–language pathologists (SLPs; mean clinical experience, 12.3 ± 7.51 years), including two doctoral-level experts with first-class clinical certification and one doctoral candidate with second-class certification. All assessments were conducted in isolated environments, and results were uploaded directly to a secure cloud-based system to ensure data confidentiality and integrity. Each evaluator assessed two voice datasets (the proposed instrument and comparator) once per participant, resulting in six independent evaluations per child (two instruments × three evaluators). This design yielded repeated observations across evaluators and instruments. Speech samples were perceptually transcribed by the SLPs, and the transcriptions were entered into the online scoring program provided with UTAP and into the Hi-DongDong digital platform, which automatically calculated the indices. The indices were calculated according to the standard UTAP scoring criteria [11,16]. The same scoring criteria were applied to both instruments.

PCC was calculated as the number of correctly produced consonants divided by the total number of target consonants × 100, with substitutions, omissions, and distortions counted as errors; PCC-R was calculated in the same manner, except that distortions were scored as correct [11,16]. Accordingly, slight distortions, such as a dentalized /s/, were counted as errors in PCC and total PCC but not in PCC-R and total PCC-R. Total PCC and total PCC-R were calculated across all consonants in the elicited words, whereas PCC and PCC-R were calculated for the designated target consonants [11,16]. PVC was calculated as the percentage of correctly produced vowels, and PWC as the percentage of words produced entirely correctly. PMLU was calculated for each word by assigning one point for each segment produced and one additional point for each correctly produced consonant, and the scores were averaged across words [11,16].

### 2.4. Data Analysis

Statistical analyses were performed using IBM SPSS Statistics (version 31.0; IBM Corp., Armonk, NY, USA), with the significance level set at *p* < 0.05. Descriptive statistics and frequency analyses were conducted to present demographic characteristics, such as age and sex, as means, standard deviations, and percentages. Inter-rater and inter-instrument reliability were evaluated using the intraclass correlation coefficient (ICC) based on a two-way mixed-effects model with absolute agreement and single measures. A mixed-effects model was used because the three evaluators and the two instruments were the specific raters and tools of interest rather than random samples from larger populations, and absolute agreement was used because the aim was to determine whether scores could be used interchangeably rather than whether they were merely consistent in rank order [18]. Under absolute agreement, the single-measure ICC is computationally identical for the two-way random- and mixed-effects models [18]. Pearson’s correlation coefficient (r) and inter-instrument ICC were calculated using the average scores of the three evaluators. As a sensitivity analysis, partial correlations controlling for age were calculated to examine whether the pooled correlations reflected age-related developmental differences. Age-stratified agreement analyses in the subset of typically developing children assessed with UTAP-2 (*n* = 505) were conducted in a separate study and are reported elsewhere [17]. Systematic errors and differences between instruments were examined using the Wilcoxon signed-rank test. Bland–Altman plots were generated to determine the mean bias, 95% limits of agreement (LoA), and corresponding 95% confidence intervals (CIs), providing a visual representation of the distribution and level of agreement between the two instruments.

## 3. Results

Between October and December 2025, 931 participants were recruited nationwide (Table 1). Because of pediatric-specific constraints, including limited attention span and difficulties coordinating follow-up sessions, not all participants completed both assessment protocols. Statistical analyses were therefore restricted to participants with valid paired datasets for each outcome measure (Table 2, Table 3 and Table 4); consequently, the effective sample size varied across analyses and is reported separately for each result.

### 3.1. Reliability

Inter-rater reliability for each instrument was ICC ≥ 0.96 across all dependent variables (Hi-DongDong, ≥ 0.99), indicating excellent reliability (*p* < 0.001) (Table 2).

Regarding inter-instrument reliability between Hi-DongDong and UTAP-2, ICC ≥ 0.75 was observed for PCC, PCC-R, total PCC, total PCC-R, and PWC, indicating good reliability; for PMLU and PVC, reliability was ICC < 0.5, indicating poor reliability (all *p* < 0.001) (Table 3). Inter-instrument reliability between Hi-DongDong and UTAP-1 was excellent for PCC, PCC-R, total PCC, and total PCC-R; reliability for PWC was good, whereas PMLU and PVC showed moderate levels (0.5 < ICC < 0.75; all *p* < 0.001) (Table 3).

### 3.2. Concurrent Validity with UTAP

Validity analysis revealed statistically significant positive correlations (*p* < 0.001) across all dependent variables for both the Hi-DongDong–UTAP-2 (*n* = 506) and Hi-DongDong–UTAP-1 (*n* = 328) comparison groups, which remained significant after adjustment for age (partial r, *p* < 0.001). Correlations between Hi-DongDong and UTAP-1 were consistently higher than those between Hi-DongDong and UTAP-2. For PCC and PCC-R, the correlation with UTAP-1 (r = 0.925; partial r = 0.924) was higher than that with UTAP-2 (r = 0.794; partial r = 0.736), and this tendency was consistently observed for total PCC and total PCC-R (UTAP-1, r = 0.932, partial r = 0.931; UTAP-2, r = 0.825, partial r = 0.771). Similarly, Hi-DongDong showed stronger correlations with UTAP-1 for both PWC (r = 0.893; partial r = 0.888) and PMLU (r = 0.939; partial r = 0.939) than with UTAP-2 (PWC, r = 0.844, partial r = 0.781; PMLU, r = 0.808, partial r = 0.752). Although PVC showed the lowest correlation coefficients overall, the correlation between Hi-DongDong and UTAP-1 (r = 0.739; partial r = 0.731) remained markedly higher than that with UTAP-2 (r = 0.347; partial r = 0.256).

The Wilcoxon signed-rank test examined systematic biases and differences between instruments. In the comparison between Hi-DongDong and UTAP-2, no significant difference was observed for total PCC and total PCC-R (*p* > 0.05), despite a tied-rank proportion of approximately 28% (Table 4). For PCC and PCC-R, despite a higher agreement rate of 44%, negative ranks were dominant, indicating significantly higher scores for Hi-DongDong (*p* < 0.05). Conversely, positive ranks were dominant for PWC, PMLU, and PVC, indicating that UTAP-2 consistently yielded significantly higher scores (*p* < 0.05). In the comparison between Hi-DongDong and UTAP-1, significant differences were observed across all dependent variables (*p* < 0.05). For PMLU, positive ranks were dominant, resulting in significantly higher UTAP-1 scores; conversely, for all other dependent variables, negative ranks were dominant, indicating that Hi-DongDong yielded relatively higher measurements (*p* < 0.05) (Table 4).

Subgroup analysis was performed on atypically developing children (*n* = 83) to evaluate clinical instrument consistency (Table 5). Consistent with the overall directional tendency, significant systematic biases were observed across all variables (*p* < 0.01). For PCC, total PCC, PWC, and PVC, negative ranks were markedly dominant, indicating that Hi-DongDong yielded significantly higher scores than UTAP-1 in this population; however, for PMLU, positive ranks were dominant, with UTAP-1 producing significantly higher values.

### 3.3. Measurement Agreement

**Figure 1:**
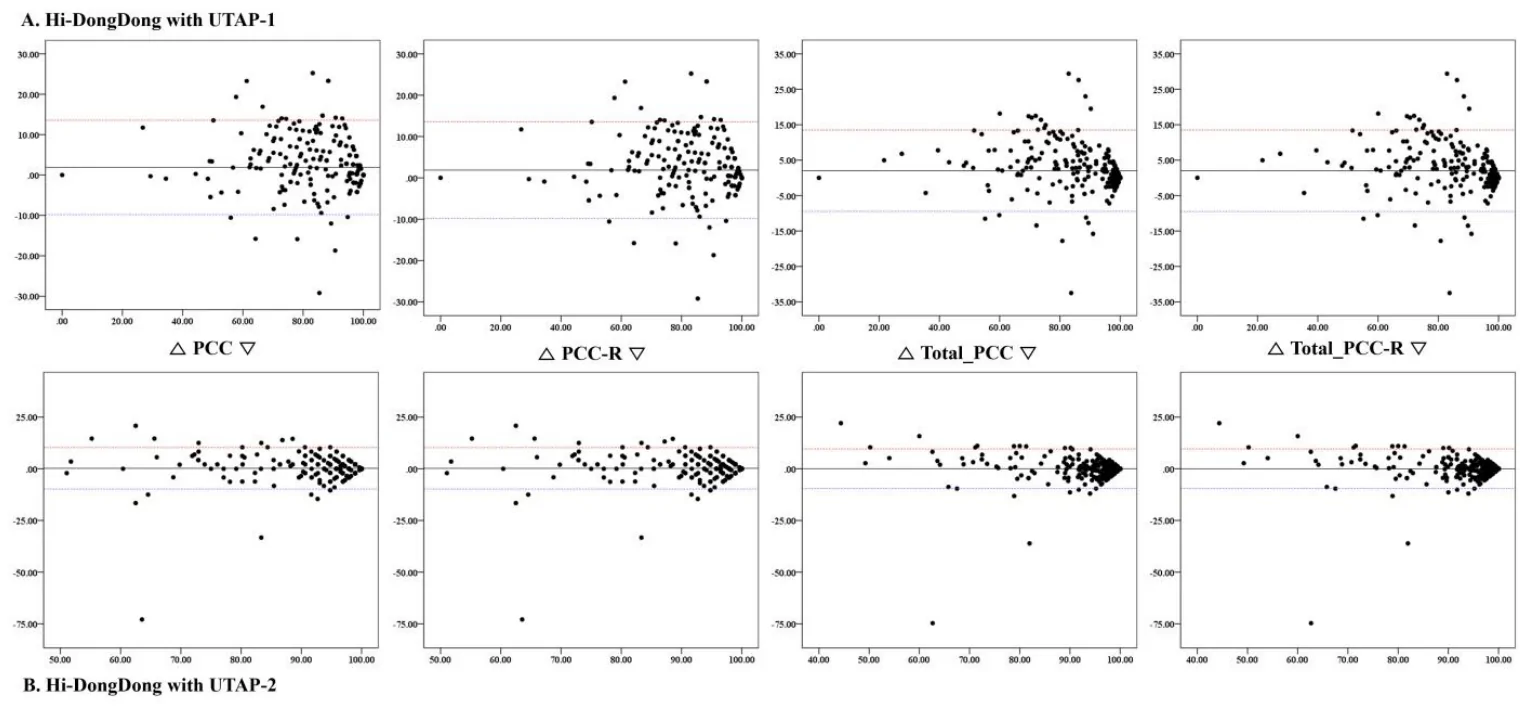
Bland–Altman plots comparing Hi-DongDong with UTAP-1 and UTAP-2 for PCC-based measures. The upper row (**A**) shows the comparison between Hi-DongDong and UTAP-1, and the lower row (**B**) shows the comparison between Hi-DongDong and UTAP-2. The four columns, from left to right, represent PCC, PCC-R, total PCC, and total PCC-R, respectively. In each plot, the x-axis represents the mean of the two assessment instruments, and the y-axis represents the difference between them (Hi-DongDong minus UTAP). The solid horizontal line indicates the mean bias, and the dashed lines represent the 95% limits of agreement. Detailed numerical values for mean bias and limits of agreement are provided in Table 6. Abbreviations: PCC, percentage of correct consonants; PCC-R, percentage of correct consonants—revised; UTAP, Urimal Test of Articulation and Phonology.

**Figure 2:**
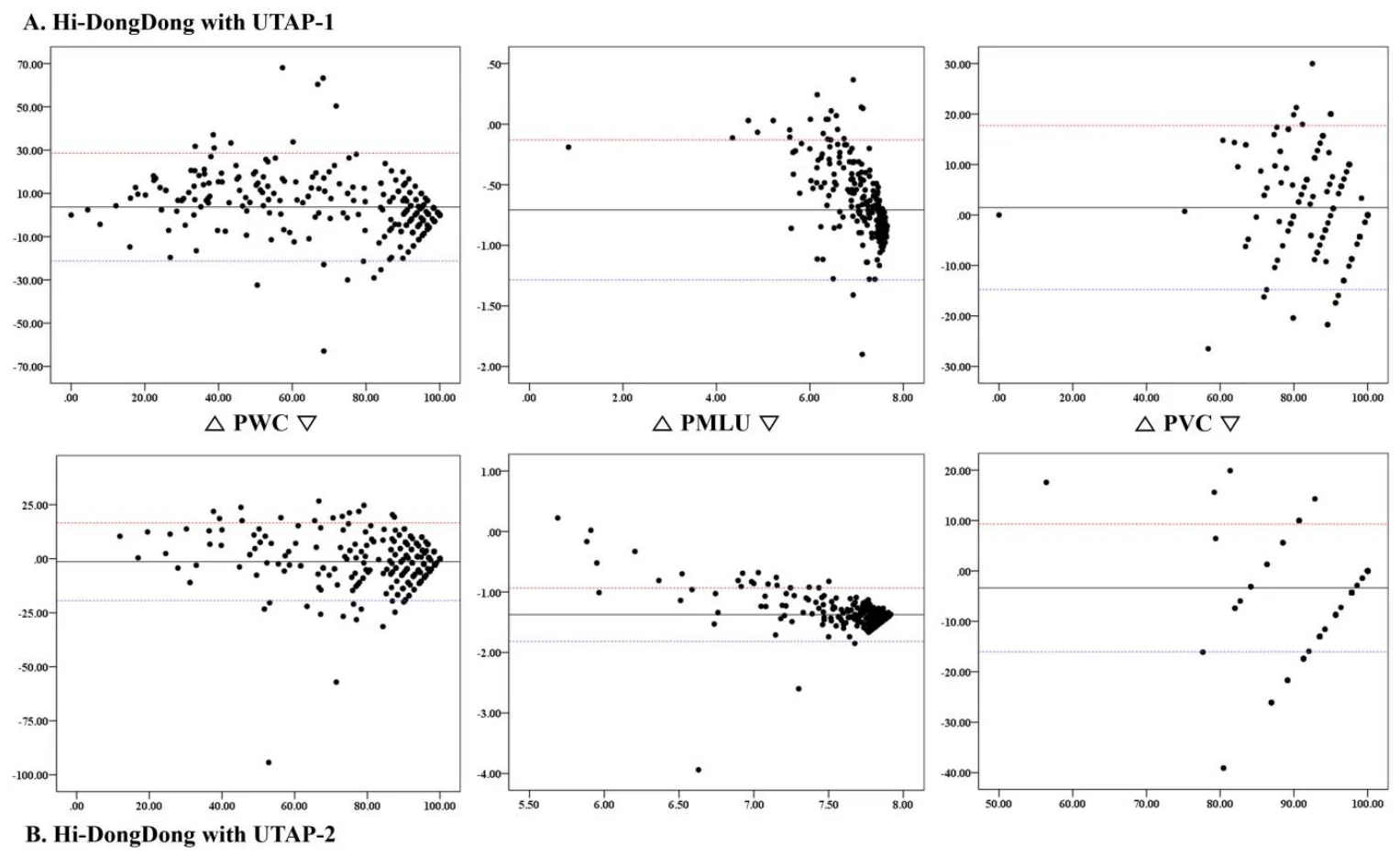
Bland–Altman plots comparing Hi-DongDong with UTAP-1 and UTAP-2 for complexity-sensitive phonological measures. The upper row (**A**) shows the comparison between Hi-DongDong and UTAP-1, and the lower row (**B**) shows the comparison between Hi-DongDong and UTAP-2. The three columns, from left to right, represent PWC, PMLU, and PVC, respectively. In each plot, the x-axis represents the mean of the two assessment instruments, and the y-axis represents the difference between them (Hi-DongDong minus UTAP). The solid horizontal line indicates the mean bias, and the dashed lines represent the 95% limits of agreement. Detailed numerical values for mean bias and limits of agreement are provided in Table 6. Abbreviations: PMLU, phonological mean length of utterance; PVC, percentage of correct vowels; PWC, percentage of correct words; UTAP, Urimal Test of Articulation and Phonology.

Bland–Altman analysis was performed to examine agreement between individual measurements obtained using the two instruments and to evaluate potential systematic bias. In the comparison between Hi-DongDong and UTAP-2 (*n* = 506), PCC, PCC-R, total PCC, and total PCC-R demonstrated minimal mean bias with symmetric LoA, indicating acceptable numerical agreement at the group level, whereas PVC showed the largest systematic bias (Table 6, Figure 1 and Figure 2). PMLU exhibited a consistent nonzero bias but a narrow LoA, suggesting high precision despite systematic differences. In the comparison between Hi-DongDong and UTAP-1 (*n* = 328), PCC-based indices showed a small positive mean bias, indicating slightly higher scores for Hi-DongDong, and PMLU demonstrated a consistent systematic bias (Table 6, Figure 1 and Figure 2). PWC demonstrated the widest LoA in both comparisons, indicating substantial variability between instruments at the individual level.

Collectively, Hi-DongDong showed a smaller mean bias and narrower LoA relative to UTAP-2 for utterance-level indices, such as total PCC and total PCC-R.

## 4. Discussion

This study aimed to evaluate the reliability and validity of Hi-DongDong, a novel digital articulation assessment instrument designed to support the digital transformation of SSD assessment in children. Using a large nationwide sample of 931 pediatric participants, this study demonstrates that Hi-DongDong exhibits excellent inter-rater reliability and strong concurrent validity with UTAP-1 and UTAP-2, particularly for consonant accuracy indices. Simultaneously, systematic differences were observed in the selected phonological indices, suggesting that although Hi-DongDong assesses constructs comparable to those of established articulation and phonology assessments, certain outcome measures may reflect instrument-specific characteristics, such as differences in item composition (e.g., the number of diphthongs) and word-level scoring structure, rather than the digital format itself, because transcription and scoring criteria were identical across instruments.

In UTAP, consonant accuracy is quantified using PCC-based indices, with the general PCC counting omissions, substitutions, and distortions as errors, and PCC-R excluding distortions from error classification [16]. Total PCC and total PCC-R emerged as the most robust outcome measures within the Hi-DongDong framework. These indices demonstrated excellent inter-instrument reliability, minimal mean bias in Bland–Altman analyses, and consistent agreement with UTAP-1 and UTAP-2, indicating strong measurement stability across assessment platforms. Speech sound production can be analyzed at multiple linguistic levels, including segmental, syllabic, word, and utterance levels [16,19]. Among these, utterance-level indices integrate phonological performance across entire speech samples, thereby reducing the influence of item-level variability and enhancing measurement stability. In pediatric populations, where attention span and task compliance may be limited, such indices offer practical advantages by maximizing reliability while minimizing testing burden. Furthermore, their favorable signal-to-noise characteristics support reliable longitudinal monitoring, reinforcing their suitability as primary outcome measures in digitally mediated assessment environments and future digital and automated interventions for SSD.

The Wilcoxon signed-rank test revealed systematic directional differences between instruments. Hi-DongDong yielded significantly higher scores than UTAP-1 across all PCC-based measures, including segment- and utterance-level indices. Compared with UTAP-2, significantly higher scores were observed only for PCC and PCC-R, whereas no significant differences were found for total PCC and total PCC-R. Compared with UTAP-2, Hi-DongDong produced lower scores on PWC, PMLU, and PVC, which capture word-level accuracy, utterance length, and phonological structural complexity, whereas compared with UTAP-1, it produced lower scores only on PMLU. These differences are best interpreted as systematic inter-instrument differences attributable to variation in analytic focus, item composition, and scoring procedures emphasized by both assessment tools. The poorer agreement observed for PWC compared with PCC is attributable to its word-level binary scoring structure, in which a single phonemic error results in credit loss for the entire word; this characteristic amplifies small phonological differences and increases inter-instrument variability, leading to wider LoA. The low correlation for PVC, particularly with UTAP-2, may be attributable to ceiling effects and to differences in diphthong composition between the instruments. Most typically developing children produced vowels with near-perfect accuracy, as reflected by the high proportion of 56% tied ranks in the Hi-DongDong–UTAP-2 comparison, which restricted score variance and attenuated the correlation. This study used a randomized allocation design, in which UTAP-1 or UTAP-2 was randomly assigned as the comparator instrument, to validate the reliability and validity of Hi-DongDong; stratified randomization based on developmental status was not implemented. Consequently, to determine whether the systematic biases observed in the total sample (typically developing, *n* = 832; atypically developing, *n* = 99) were inherent to the tools or an artifact of ceiling effects within the predominantly typical sample, a subgroup analysis was conducted in atypically developing children (*n* = 83). In this subgroup, comparison with UTAP-2 was constrained because most participants underwent UTAP-1 assessment. Nevertheless, Hi-DongDong yielded significantly higher measurements across all variables except PMLU, consistent with the primary analysis. The persistence of these disparities in the atypically developing subgroup—where scores are distributed substantially below the ceiling—suggests that the scoring gaps are not attributable to scale attenuation or artifacts of sample distribution. Although this subgroup was clinically heterogeneous and too small for diagnosis-specific analyses, the consistency of these findings suggests that the observed differences are more likely related to instrument characteristics than to the score distribution of the typically developing sample. The range restriction inherent in the sample likely influenced the magnitude of the reported validity coefficients. Although correlation coefficients were higher for the Hi-DongDong–UTAP-1 comparison, which benefited from range enhancement through inclusion of a broader clinical spectrum, correlations with UTAP-2 remained robust despite restricted score variance within the predominantly typically developing population [20]. The sustained high degree of agreement across both comparison groups, regardless of sample homogeneity, reinforces the concurrent validity and evaluative stability of Hi-DongDong as a reliable assessment tool across varying levels of phonological proficiency.

Bland–Altman analyses indicated that the systematic bias for PMLU was consistent in direction across both comparisons, whereas the direction of bias for PVC differed between the UTAP-1 and UTAP-2 comparisons; the consistent PMLU bias supports the interpretation of predictable instrument-specific characteristics rather than random measurement error. In contrast, utterance-level consonant accuracy indices demonstrated minimal mean bias and relatively narrow LoA compared with UTAP-2, consistent with the absence of significant differences in the Wilcoxon signed-rank test. Furthermore, Hi-DongDong is designed to support digital data processing and structured analysis in clinical settings. Although speech samples were perceptually transcribed by trained SLPs in the present study, as described in Section 2.3, the structured digital format of Hi-DongDong has the potential to reduce reliance on time-intensive manual transcription and token-level scoring through future integration with automated systems. This design aligns with utterance-level PCC-based measures, which prioritize global speech accuracy over exhaustive token-level error analysis. Collectively, these findings suggest that discrepancies observed across specific measures are better interpreted as reflecting differences in analytic focus and item structure rather than measurement error. The strong agreement observed for utterance-level PCC indices, combined with the structured and digital-compatible design of Hi-DongDong, supports its concurrent validity with established instruments for assessing consonant accuracy in children.

Age-stratified analyses in typically developing children assessed with UTAP-2 have been reported separately [17]; these showed comparable developmental trajectories between the instruments, with narrow LoA in school-age children and wider LoA in preschool-age children. Age-stratified analyses for the UTAP-1 comparison and for atypically developing children were not performed because of limited sample sizes in each age group.

### Limitations

This study had several limitations. First, intra-rater reliability could not be assessed because of the temporal and physical constraints involved in evaluating a large-scale sample. Second, the participant pool predominantly comprised typically developing children, which may limit the generalizability of the findings to atypically developing children with lower articulation proficiency. In addition, the atypically developing subgroup comprised clinically heterogeneous conditions with small diagnostic categories; therefore, the subgroup findings should not be generalized to any specific clinical population. Third, this study assessed concurrent validity and inter-instrument agreement but not diagnostic accuracy (e.g., sensitivity, specificity, or ROC analysis). Therefore, Hi-DongDong cannot currently replace the diagnostic thresholds of existing standardized instruments, and its potential role as a screening and monitoring tool should be confirmed in diagnostic accuracy studies after age-specific norms are established. Fourth, the order of administration of the two instruments was fixed and was neither counterbalanced nor randomized. Because both instruments were administered consecutively in a single session, fatigue, reduced attention, or practice effects, particularly in younger children with limited attention spans, may have influenced performance on the instrument administered second. Therefore, the systematic differences observed between the instruments may partly reflect order effects, which could not be separated from instrument-specific characteristics in the present design. Future studies should counterbalance the order of administration. Finally, because distinct systematic biases were observed between the instruments, the diagnostic thresholds of the existing standardized tools could not be directly applied. Moreover, age-specific normative data for Hi-DongDong are not yet available; therefore, caution is required when interpreting results for clinical purposes. Development of age-specific norms for Hi-DongDong should be prioritized in future research.

## 5. Conclusions

Hi-DongDong demonstrated excellent inter-rater reliability and good agreement with established standardized instruments. These findings support the reliability and concurrent validity of Hi-DongDong, particularly for utterance-level indices. At present, Hi-DongDong should be regarded as a complementary tool with potential for screening and monitoring rather than as a replacement for standardized diagnostic assessment; age-specific norms and diagnostic accuracy studies are required before its clinical application.

## Figures and Tables

**Table 1:** Baseline characteristics of the study participants.

Characteristic	Category	Value [*n* (%)]
Sex	Male	490 (52.63)
	Female	441 (47.37)
Age	Toddlerhood, 2 years	44 (4.73)
	Preschool age, 3–5 years	208 (22.34)
	Early school age, 6–9 years	439 (47.15)
	Late school age, 10–13 years	240 (25.78)
Geographic location	Seoul Metropolitan Area	376 (40.39)
	Central Region	109 (11.71)
	Southeast Region	371 (39.85)
	Southwest Region	73 (7.84)
	Others	2 (0.21)
Underlying condition	Typically developing	832 (89.37)
	At risk for developmental delay	1 (0.11)
	Developmental delay	11 (1.18)
	Developmental disability	31 (3.33)
	Autism spectrum disorder	22 (2.36)
	Intellectual disability	32 (3.44)
	Brain lesion disorder	2 (0.21)

**Table 2:** Inter-rater reliability for each assessment instrument.

Variable	Instrument	N	ICC	95% CI	F (df1, df2)	*p*
PCC	Hi-DongDong	871	0.999	[0.998, 0.999]	2060.39 (870, 1740)	<0.001
	UTAP-2	551	0.998	[0.998, 0.998]	1627.41 (550, 1100)	<0.001
	UTAP-1	337	0.985	[0.982, 0.988]	200.04 (336, 672)	<0.001
PCC-R	Hi-DongDong	871	0.999	[0.998, 0.999]	2084.03 (870, 1740)	<0.001
	UTAP-2	551	0.998	[0.998, 0.999]	1917.74 (550, 1100)	<0.001
	UTAP-1	337	0.985	[0.982, 0.988]	201.99 (336, 672)	<0.001
Total PCC	Hi-DongDong	871	0.999	[0.999, 0.999]	2867.72 (870, 1740)	<0.001
	UTAP-2	551	0.998	[0.998, 0.999]	1899.14 (550, 1100)	<0.001
	UTAP-1	337	0.983	[0.979, 0.986]	171.90 (336, 672)	<0.001
Total PCC-R	Hi-DongDong	871	0.999	[0.999, 0.999]	2930.42 (870, 1740)	<0.001
	UTAP-2	551	0.999	[0.999, 0.999]	2973.20 (550, 1100)	<0.001
	UTAP-1	337	0.983	[0.979, 0.986]	172.59 (336, 672)	<0.001
PWC	Hi-DongDong	871	0.998	[0.998, 0.998]	1729.37 (870, 1740)	<0.001
	UTAP-2	551	0.998	[0.998, 0.999]	1958.90 (550, 1100)	<0.001
	UTAP-1	337	0.997	[0.996, 0.997]	936.91 (336, 672)	<0.001
PMLU	Hi-DongDong	871	0.999	[0.999, 0.999]	4831.67 (870, 1740)	<0.001
	UTAP-2	551	0.999	[0.999, 0.999]	2387.78 (550, 1100)	<0.001
	UTAP-1	337	0.987	[0.984, 0.989]	227.61 (336, 672)	<0.001
PVC	Hi-DongDong	871	0.996	[0.995, 0.996]	664.87 (870, 1740)	<0.001
	UTAP-2	551	0.986	[0.984, 0.988]	210.00 (550, 1100)	<0.001
	UTAP-1	337	0.969	[0.962, 0.974]	93.15 (336, 672)	<0.001

CI, confidence interval; df, degrees of freedom; F (df1, df2), F-statistic of the F-test for the null hypothesis ICC = 0, with numerator (df1) and denominator (df2) degrees of freedom; ICC, intraclass correlation coefficient; PCC, percentage of correct consonants; PCC-R, percentage of correct consonants—revised; PMLU, phonological mean length of utterance; PVC, percentage of correct vowels; PWC, percentage of correct words; UTAP, Urimal Test of Articulation and Phonology.

**Table 3:** Inter-instrument reliability.

Variable	Comparison	N	ICC (3,1)	95% CI	F (df1, df2)	*p*
PCC	Hi-DongDong–UTAP-2	506	0.794	[0.759, 0.824]	8.70 (505, 505)	<0.001
	Hi-DongDong–UTAP-1	328	0.916	[0.885, 0.937]	24.96 (327, 327)	<0.001
PCC-R	Hi-DongDong–UTAP-2	506	0.794	[0.760, 0.824]	8.72 (505, 505)	<0.001
	Hi-DongDong–UTAP-1	328	0.916	[0.885, 0.938]	24.97 (327, 327)	<0.001
Total PCC	Hi-DongDong–UTAP-2	506	0.824	[0.794, 0.850]	10.36 (505, 505)	<0.001
	Hi-DongDong–UTAP-1	328	0.920	[0.886, 0.942]	26.54 (327, 327)	<0.001
Total PCC-R	Hi-DongDong–UTAP-2	506	0.824	[0.794, 0.851]	10.38 (505, 505)	<0.001
	Hi-DongDong–UTAP-1	328	0.920	[0.886, 0.942]	26.50 (327, 327)	<0.001
PWC	Hi-DongDong–UTAP-2	506	0.840	[0.812, 0.865]	11.76 (505, 505)	<0.001
	Hi-DongDong–UTAP-1	328	0.876	[0.836, 0.905]	16.29 (327, 327)	<0.001
PMLU	Hi-DongDong–UTAP-2	506	0.077	[−0.013, 0.289]	7.38 (505, 505)	<0.001
	Hi-DongDong–UTAP-1	328	0.593	[−0.064, 0.864]	20.67 (327, 327)	<0.001
PVC	Hi-DongDong–UTAP-2	506	0.264	[0.121, 0.387]	1.91 (505, 505)	<0.001
	Hi-DongDong–UTAP-1	328	0.729	[0.671, 0.777]	6.54 (327, 327)	<0.001

CI, confidence interval; df, degrees of freedom; F (df1, df2), F-statistic of the F-test for the null hypothesis ICC = 0, with numerator (df1) and denominator (df2) degrees of freedom; ICC, intraclass correlation coefficient; PCC, percentage of correct consonants; PCC-R, percentage of correct consonants—revised; PMLU, phonological mean length of utterance; PVC, percentage of correct vowels; PWC, percentage of correct words; UTAP, Urimal Test of Articulation and Phonology.

**Table 4:** Comparison of scores and assessment of systematic bias between the two instruments.

Comparison (N)	Variable	Negative Ranks	Positive Ranks	Ties (%)	Z	*p*
Hi-DongDong–UTAP-2 (506)	PCC	162	121	223 (44)	−2.349	0.019
	PCC-R	162	121	223 (44)	−2.287	0.022
	Total PCC	179	187	140 (28)	−0.327	0.744
	Total PCC-R	179	188	139 (28)	−0.206	0.837
	PWC	166	251	89 (18)	−3.995	<0.001
	PMLU	2	504	0 (0)	−19.518	<0.001
	PVC	16	208	282 (56)	−10.899	<0.001
Hi-DongDong–UTAP-1 (328)	PCC	160	78	90 (27)	−6.242	<0.001
	PCC-R	161	77	90 (27)	−6.209	<0.001
	Total PCC	179	89	60 (18)	−6.988	<0.001
	Total PCC-R	179	89	60 (18)	−6.975	<0.001
	PWC	197	92	39 (12)	−5.768	<0.001
	PMLU	11	317	0 (0)	−15.612	<0.001
	PVC	118	104	106 (32)	−3.228	0.001

Z, standardized test statistic of the Wilcoxon signed-rank test. Negative ranks indicate cases in which the UTAP score was lower than the Hi-DongDong score; positive ranks indicate cases in which the UTAP score was higher than the Hi-DongDong score.PCC, percentage of correct consonants; PCC-R, percentage of correct consonants—revised; PMLU, phonological mean length of utterance; PVC, percentage of correct vowels; PWC, percentage of correct words; UTAP, Urimal Test of Articulation and Phonology.

**Table 5:** Comparison of scores and assessment of systematic bias in atypically developing children.

Comparison (N)	Variable	Negative Ranks	Positive Ranks	Ties (%)	Z	*p*
Hi-DongDong–UTAP-1 (83)	PCC	61	20	2 (2.41)	−4.169	<0.001
	PCC-R	61	20	2 (2.41)	−4.169	<0.001
	Total PCC	68	14	1 (1.2)	−5.832	<0.001
	Total PCC-R	68	14	1 (1.2)	−5.832	<0.001
	PWC	67	15	1 (1.2)	−5.832	<0.001
	PMLU	6	77	0 (0)	−7.714	<0.001
	PVC	48	32	3 (3.61)	−3.065	0.002

The atypically developing subgroup (*n* = 83) comprised children with developmental delay (*n* = 10), developmental disability (*n* = 27), autism spectrum disorder (*n* = 20), intellectual disability (*n* = 24), and brain lesion disorder (*n* = 2). Of the 99 atypically developing children, 16 who did not complete the UTAP-1 assessment were excluded. PCC, percentage of correct consonants; PCC-R, percentage of correct consonants—revised; PMLU, phonological mean length of utterance; PVC, percentage of correct vowels; PWC, percentage of correct words; UTAP, Urimal Test of Articulation and Phonology.

**Table 6:** Mean bias and 95% limits of agreement between the two assessment instruments.

Variable	Comparison	Mean Bias ± SE [95% CI]	Upper LoA [95% CI]	Lower LoA [95% CI]
PCC	Hi-DongDong–UTAP-1	1.88 ± 0.33 [1.23, 2.53]	10.28 [9.71, 10.85]	−9.81 [−10.38, −9.24]
	Hi-DongDong–UTAP-2	0.24 ± 0.23 [−0.21, 0.68]	13.55 [13.15, 13.95]	−9.79 [−10.19, −9.39]
PCC-R	Hi-DongDong–UTAP-1	1.88 ± 0.33 [1.23, 2.52]	10.26 [9.69, 10.83]	−9.81 [−10.38, −9.24]
	Hi-DongDong–UTAP-2	0.23 ± 0.23 [−0.22, 0.67]	13.55 [13.15, 13.95]	−9.80 [−10.20, −9.40]
Total PCC	Hi-DongDong–UTAP-1	2.01 ± 0.32 [1.37, 2.65]	9.60 [9.05, 10.15]	−9.61 [−10.16, −9.06]
	Hi-DongDong–UTAP-2	−0.00 ± 0.22 [−0.43, 0.42]	13.49 [13.11, 13.87]	−9.47 [−9.85, −9.09]
Total PCC-R	Hi-DongDong–UTAP-1	2.01 ± 0.32 [1.37, 2.64]	9.58 [9.03, 10.13]	−9.61 [−10.16, −9.06]
	Hi-DongDong–UTAP-2	−0.02 ± 0.22 [−0.44, 0.41]	13.50 [13.12, 13.88]	−9.48 [−9.86, −9.10]
PWC	Hi-DongDong–UTAP-1	3.72 ± 0.70 [2.34, 5.10]	16.62 [15.41, 17.83]	−19.41 [−20.62, −18.20]
	Hi-DongDong–UTAP-2	−1.39 ± 0.41 [−2.20, −0.59]	28.64 [27.93, 29.35]	−21.20 [−21.91, −20.49]
PMLU	Hi-DongDong–UTAP-1	−0.71 ± 0.02 [−0.74, −0.68]	−0.93 [−0.96, −0.90]	−1.82 [−1.85, −1.79]
	Hi-DongDong–UTAP-2	−1.38 ± 0.01 [−1.40, −1.36]	−0.13 [−0.15, −0.11]	−1.29 [−1.31, −1.27]
PVC	Hi-DongDong–UTAP-1	1.49 ± 0.46 [0.59, 2.39]	9.30 [8.50, 10.10]	−16.04 [−16.84, −15.24]
	Hi-DongDong–UTAP-2	−3.37 ± 0.29 [−3.93, −2.80]	17.72 [17.22, 18.22]	−14.75 [−15.25, −14.25]

CI, confidence interval; LoA, limit of agreement; PCC, percentage of correct consonants; PCC-R, percentage of correct consonants—revised; PMLU, phonological mean length of utterance; PVC, percentage of correct vowels; PWC, percentage of correct words; UTAP, Urimal Test of Articulation and Phonology.

## Data Availability

The data presented in this study are available on request from the corresponding author, in accordance with the approved IRB protocol and applicable privacy protections for pediatric participants.

## Supplementary Materials

The following supporting information can be downloaded at: https://www.mdpi.com/article/10.3390/children13101394/s1, Supplementary Material S1: Hi-DongDong Assessment Protocol, Stimuli, and Scoring Methodology.

## Abbreviations

The following abbreviations are used in this manuscript:

SSD	speech sound disorder(s)
UTAP	Urimal Test of Articulation and Phonology
ICC	intraclass correlation coefficient
AI	artificial intelligence
SLP	speech–language pathologist
PCC	percentage of correct consonants
PCC-R	percentage of correct consonants–revised
PMLU	phonological mean length of utterance
PVC	percentage of correct vowels
PWC	percentage of correct words
LoA	limit of agreement
CI	confidence interval
