# Supplementary material for: Digital Transformation of Korean Articulation and Phonology Assessment: Reliability and Validity of Hi-DongDong
Source: Children (Basel). 2026 Oct 13;13(10):1394. doi: 10.3390/children13101394 (PMC13703450; doi:10.3390/children13101394)
Supplement: Supplementary file 1 [file children-13-01394-s001.zip › children-4596763-supplementary.pdf]

Supplementary Material S1. Hi-DongDong Assessment Protocol, Stimuli, and Scoring Methodology

Assessment Environment

The assessment is conducted in a quiet, independent space where external noise is strictly controlled, allowing the child to remain fully engaged and focused on the task.

Stimulus Composition and Administration Procedure

The assessment is conducted using 35 target words presented in [Table 1]. To facilitate the child's understanding, each target word is provided simultaneously with its corresponding visual stimulus (illustration) and text.

The assessment is administered in stages based on the child's response, as follows:

1) Spontaneous Naming Stage: The child is encouraged to name the target word spontaneously upon seeing the presented illustration or text.

2) Imitative Naming Stage: In cases where the child is unable to read the text, fails to recognize the word from the illustration alone, or does not produce a spontaneous response, the examiner provides a standard pronunciation of the target word and asks the child to imitate it directly.

Transcription and Scoring Method

The examiner transcribes the child's target vocalizations through perceptual assessment. Subsequently, the data is analyzed and calculated based on the phonemic composition of each category specified in [Table 2]. The distribution of target phonemes by word position and the syllable structures represented in the word list are summarized in [Table 3].

Table S1. Target Word List (35 Items)

Target words	Meaning	IPAc	Target words	Meaning	IPAc
타조	ostrich	[tʰa̱.d͡ʑo̝]	빵	bread	[p͈a̱ŋ]
냄비	pot	[nɛm.bi]	아빠	dad	[a̱p͈a̱]
눈썹	eyebrow	[nun.s͈ʌp̚]	책상	desk	[t͡ɕʰɛk̚.s͈a̱ŋ]
딸기	Strawberry	[t͈a̱l.gi]	찌개	stew	[t͡ɕ͈i.gɛ]
국자	ladle	[kuk̚.t͡ɕ͈a̱]	오뚝이	roly-poly toy	[o̝.t͈u.gi]
다리	leg/bridge	[ta̱.ɾi]	하마	hippopotamus	[ha̱.ma̱]
자동차	car	[t͡ɕa̱.do̝ŋ.t͡ɕʰa̱]	구급차	ambulance	[ku.gɯp̚.t͡ɕʰa̱]
씨앗	seed	[ɕ͈i.a̱t̚]	수박	watermelon	[su.ba̱k̚]
바나나	banana	[pa̱.na̱.na̱]	과자	snack	[kwa̱.d͡ʑa̱]
레몬	lemon	[ɾe̝.mo̝n]	돼지	pig	[twe̝.d͡ʑi]
코끼리	elephant	[kʰo̝.k͈i.ɾi]	요거트	yogurt	[jo̝.gʌ.tʰɯ]
곰	bear	[ko̝m]	귀	ear	[kɥi]
사탕	candy	[sa̱.tʰa̱ŋ]	여우	fox	[jʌ.u]
버섯	mushroom	[pʌ.sʌt̚]	우유	milk	[u.ju]
펭귄	penguin	[pʰe̝ŋ.gɥin]	야구	baseball	[ja̱.gu]
마이크	microphone	[ma̱.i.kʰɯ]	원숭이	monkey	[wʌn.su.ŋi]
연필	pencil	[jʌn.pʰil]	의자	chair	[ɰi.d͡ʑa̱]
까치	magpie	[k͈a̱.t͡ɕʰi]			

Table S2. Distribution of Target Phonemes by Category

Category	Subcategory	Denominator	Unit
Accuracy	Consonant Accuracy (PCC)	/48	%
	Revised Consonant Accuracy (PCC-Revised)	/48	%
	Total Consonant Accuracy (Total PCC)	/83	%
	Total Revised Consonant Accuracy (Total PCC-Revised)	/83	%
	Vowel Accuracy (PVC)	/14	%
	Diphthong Accuracy	/11	%
	Word Accuracy (PWC)	/35	%
	Phonological Mean Length of Utterance (PMLU)	-	Mean score
Articulation Manner	Plosive	/23	%
	Fricative	/5	%
	Affricate	/6	%
	Nasal	/10	%
	Liquid	/4	%
Articulation Place	Bilabial	/12	%
	Alveolar	/19	%
	Palatal	/6	%
	Velar	/10	%
	Glottal	/1	%
Phonological Features	Continuancy	/6	%
	Delayed Release	/6	%
	Laterality	/3	%
	Coronal	/23	%
	Anterior	/27	%
	Tenseness	/18	%
	Aspiration	/8	%
	High Tongue	/14	%
	Back Tongue	/10	%
	Roundedness	/3	%
	High	/6	%
	Low	/1	%
	Back	/7	%
Error Patterns – Substitution	Plosivization	/21	%
	Fricativization	/37	%
	Affricativization	/37	%
	Nasalization	/36	%
	Liquidization	/40	%
	Bilabialization	/33	%
	Alveolarization	/26	%
	Palatalization	/37	%
	Velarization	/35	%
	Glottalization	/41	%
	Tense Consonantization	/34	%
	Aspirated Consonantization	/33	%
Error Patterns – Assimilation	Plosive Assimilation	/16	%
	Fricative Assimilation	/5	%
	Affricate Assimilation	/8	%
	Nasal Assimilation	/12	%
	Liquid Assimilation	/9	%
	Bilabial Assimilation	/11	%
	Alveolar Assimilation	/13	%
	Palatal Assimilation	/8	%
	Velar Assimilation	/14	%
	Glottal Assimilation	/2	%
	Tense Assimilation	/9	%
	Aspirated Assimilation	/9	%

Table S3. Distribution of Target Phonemes and Syllable Structures by Word Position in the Hi-DongDong Word List

Phoneme	Word-initial	Word-medial	Syllable-final (coda)
**A. Consonants (19)**			
ㄱ /k/	국자, 곰, 구급차, 과자, 귀	딸기, 찌개, 오뚝이, 구급차, 펭귄, 요거트, 야구	국자, 책상, 수박
ㄲ /k͈/	까치	코끼리	–
ㅋ /kʰ/	코끼리	마이크	–
ㄷ /t/	다리, 돼지	자동차	씨앗, 버섯
ㄸ /t͈/	딸기	오뚝이	–
ㅌ /tʰ/	타조	사탕, 요거트	–
ㅂ /p/	바나나, 버섯	냄비, 수박	눈썹, 구급차
ㅃ /p͈/	빵	아빠	–
ㅍ /pʰ/	펭귄	연필	–
ㅈ /tɕ/	자동차	타조, 과자, 돼지, 의자	–
ㅉ /tɕ͈/	찌개	국자*	–
ㅊ /tɕʰ/	책상	자동차, 까치, 구급차	–
ㅅ /s/	사탕, 수박	버섯, 원숭이	–
ㅆ /s͈/	씨앗	눈썹, 책상*	–
ㅎ /h/	하마	–	–
ㅁ /m/	마이크	레몬, 하마	냄비, 곰
ㄴ /n/	냄비, 눈썹	바나나	눈썹, 레몬, 펭귄, 연필, 원숭이
ㅇ /ŋ/	n/a†	–	자동차, 사탕, 펭귄, 빵, 책상, 원숭이‡
ㄹ /l/, [ɾ]	레몬	다리, 코끼리	딸기, 연필
**B. Monophthongs (7/7)**			
ㅏ /a/	타조, 다리, 바나나, 사탕, 까치, 하마, 빵		
ㅓ /ʌ/	눈썹, 버섯, 요거트		
ㅗ /o/	타조, 자동차, 레몬, 코끼리, 곰, 오뚝이		
ㅜ /u/	눈썹, 국자, 오뚝이, 구급차, 수박, 여우, 야구		
ㅡ /ɯ/	구급차, 요거트, 마이크		
ㅣ /i/	냄비, 딸기, 다리, 씨앗, 코끼리, 까치, 돼지		
ㅔ/ㅐ /e/§	냄비, 레몬, 책상, 찌개, 펭귄		
**C. Diphthongs (9/10)**			
ㅑ /ja/	야구		
ㅕ /jʌ/	연필, 여우		
ㅛ /jo/	요거트		
ㅠ /ju/	우유		
ㅘ /wa/	과자		
ㅙ/ㅚ/ㅞ /we/§	돼지		
ㅝ /wʌ/	원숭이		
ㅟ /wi/ [ɥi]	귀, 펭귄		

Phoneme	Word-initial	Word-medial	Syllable-final (coda)
ㅢ /ɰi/	의자		
ㅖ/ㅒ /je/	Not included		
**D. Syllable structures (8/8)**			
V	아 (아빠), 오 (오뚝이), 이 (마이크), 우 (여우, 우유)		
CV	타·조 (타조), 다·리 (다리), 바·나·나 (바나나)		
VC	앗 (씨앗)		
CVC	곰, 빵, 냄 (냄비), 눈·썹 (눈썹), 국 (국자), 박 (수박)		
GV	여 (여우), 요 (요거트), 유 (우유), 야 (야구), 의 (의자)		
CGV	과 (과자), 돼 (돼지), 귀		
GVC	연 (연필), 원 (원숭이)		
CGVC	귄 (펭귄)		

Positions are based on the surface pronunciation shown in Table 1. Word-medial refers to syllable-initial position within a word (non-initial syllable).

*Realized as a fortis consonant through post-obstruent tensification (국자 [kuk̚.t͡ɕ͈a], 책상 [t͡ɕʰɛk̚.s͈aŋ]).

†ㅇ /ŋ/ does not occur in syllable-initial position in Korean. ‡Phonologically a coda of 숭; resyllabified as the onset of the following vowel in Table 1 ([wʌn.su.ŋi]).

Only seven consonants (/p, t, k, m, n, ŋ, l/) occur in syllable-final position; − indicates a position not permitted by Korean phonotactics or not represented in the word list (ㅎ in word-medial position).

§/e/ and /ɛ/ (ㅔ, ㅐ) and /we/ (ㅙ, ㅚ, ㅞ) are merged in contemporary Seoul Korean.

C, consonant; V, vowel; G, glide.
